# Review of PD-1/PD-L1 Inhibitors in Metastatic dMMR/MSI-H Colorectal Cancer

**DOI:** 10.3389/fonc.2019.00396

**Published:** 2019-05-14

**Authors:** André F. Oliveira, Luís Bretes, Irene Furtado

**Affiliations:** Serviço de Oncologia Médica, Centro Hospitalar Universitário Algarve, Faro, Portugal

**Keywords:** immunotherapy, colorectal cancer, PD-1, inhibitors, PD-L1, microsatellite instability, MSI-H, dMMR

## Abstract

There are a wide range of therapies for metastatic colorectal cancer (CRC) available, but outcomes remain suboptimal. Learning the role of the immune system in cancer development and progression led to advances in the treatment over the last decade. While the field is rapidly evolving, PD-1, and PD-L1 inhibitors have a leading role amongst immunomodulatory agents. They act against pathways involved in adaptive immune suppression resulting in immune checkpoint blockade. Immunotherapy has been slow to impact the management of this patient population due to disappointing results, mainly when used broadly. Nevertheless, some patients with microsatellite-instability-high (MSI-H) or mismatch repair-deficient (dMMR) CRC appear to be susceptible to checkpoint inhibitors with objective and sustained clinical responses, providing a new therapeutic option for patients with advanced disease. This article provides a comprehensive review of the early and late phase trials with the updated data of PD-1/PD-L1 inhibitors alone or in combination with other therapies (immunotherapy, targeted therapy and chemotherapy). While data is still limited, many ongoing trials are underway, testing the efficacy of these agents in CRC. Current and future challenges of PD-1 and PD-L1 inhibitors are also discussed.

## Introduction

Colorectal cancer (CRC) is the third most common cancer in men and the second in women worldwide. The incidence across the globe is different in various countries, but about 55% of the cases occur in more developed regions. Despite this fact, geographic patterns are very similar in men and women (1). Estimates indicate that nearly half of patients with CRC are found with hepatic metastasis during the follow-up of their disease (2). Regrettably, most patients won't undergo surgery for metastases because of unresectability or comorbidities. Clinical outcomes have however improved over the past two decades. Chemotherapy with biological agents remains the standard of care of today. Patients with metastatic colorectal cancer (mCRC) have a median overall survival (OS) of 26 months for RAS-mutated and 30 months for those with RAS wild-type status (3, 4).

Recently immunotherapy changed the treatment paradigm and its respective outcomes in many tumor types like melanoma, renal, bladder and lung cancer.

### Immunotherapy With PD-1 Inhibition

**Figure 1:**
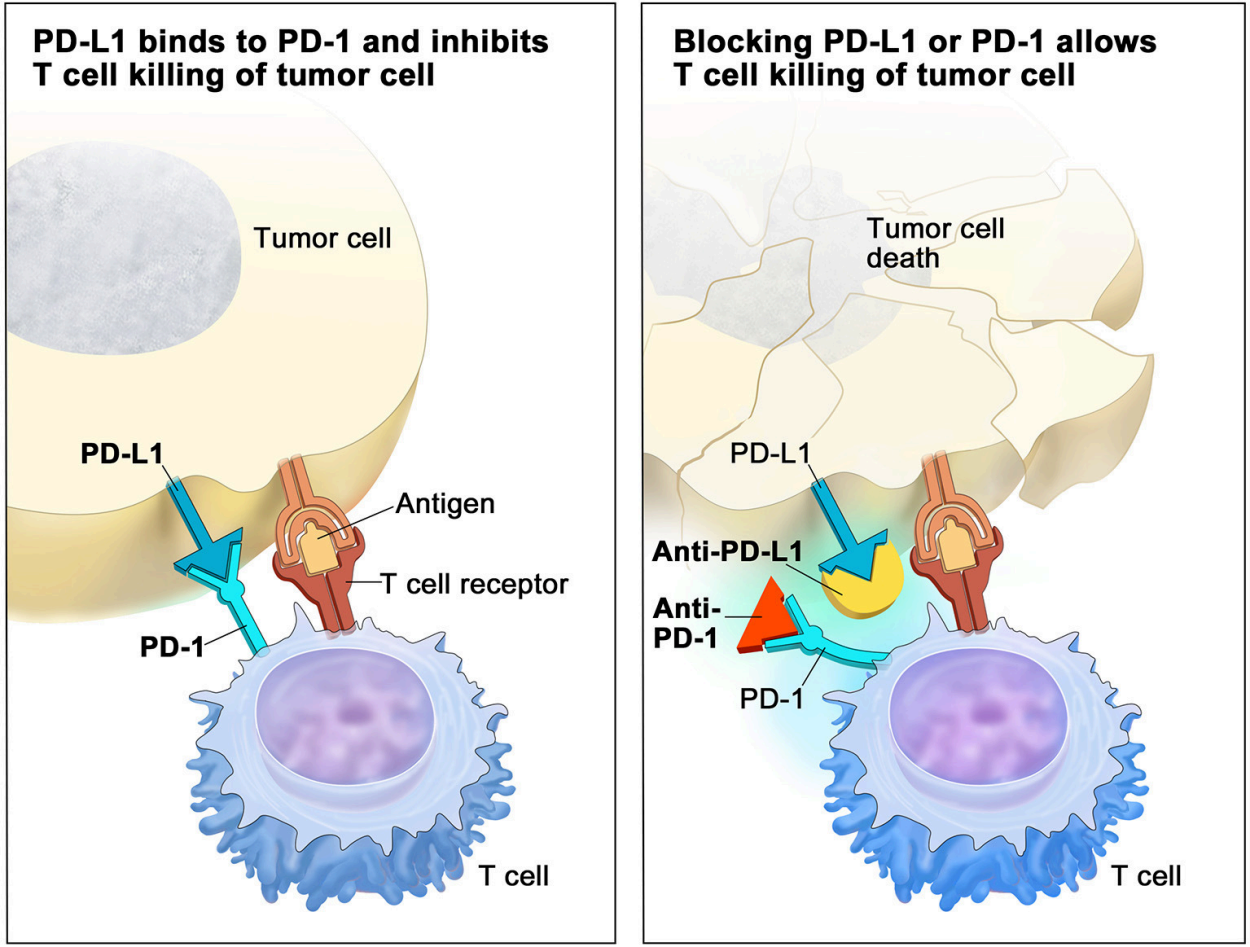
Deactivated T Cell **(Left)**: when programmed-death receptor (PD-1) on the T cell binds to programmed death-ligand 1 (PD-L1) on the tumor cell, the T cell becomes deactivated, allowing the cancer cell to evade immune attack. Inhibitors of PD-1 and PD-L1 can prevent the tumor cell from binding to PD-1, enabling the T cell to remain active and co-ordinate an attack (on the **Right**). Reproduced with permission of Terese Winslow LLC. Credit: For the National Cancer Institute © 2015 Terese Winslow LLC, U.S. Govt. has certain rights.

Nobel Prize in Physiology or Medicine of 2018 was awarded for the discovery of cancer therapy by the inhibition of negative immune regulation. Research lead by Prof. Honjo identified PD-1 (Programmed cell death protein-1) as a component of inhibition of the immune system, noting that disruption of PD-1 in pre-clinical models resulted in increased immune system activity. This key information helped initiate the development of anti-PD-1/PD-L1 (Programmed death ligand-1) checkpoint inhibitors that are now a standard of care treatments for several tumor types (5). PD-1 is a cell surface receptor found on activated T cells, pro-B cells and macrophages. When PD-L1 is bound to PD-1, the result is a counter-inhibitory negative feedback loop. This functions as a protective mechanism that prevents a host from an attack by its own immune system (6, 7). Some cancers exploit this negative feedback loop by making its cells express PD-L1 in order to evade immunosurveillance. Binding of PD-1 to PD-L1 was shown to disable the effector function of lymphocytes, decrease T cell receptor-mediated activation and exhaust proliferation of cytotoxic T lymphocytes in response to cancer cells. This results in impaired immune activity known as T-cell exhaustion (8). T cells can remain in a state of anergy (9) and cancer cells escape immune surveillance. The goal of PD-1/PD-L1 inhibitors is to block this inhibitory immune checkpoint molecule (Figure 1). Results of this therapy are primarily unknown in CRCs.

### Mismatch Repair Deficient (dMMR)/Microsatellite Instability-High (MSI-H) Tumors

Findings of previous studies with PD-1 inhibitors in mCRC were not notably encouraging. Responses were only rarely seen (10). Further interpretation of these studies revealed a high tumor mutational burden (TMB) as a predictive marker for response. The rationale was that the higher the TMB, the higher neo-antigen load and therefore higher tumor immunogenicity (11). Also of interest were findings of types of mutations that would determine neo-antigen load. It was found the greatest with by insertion or deletions (12). The population of patients with CRC with these characteristics are relatively small and are mainly constituted by tumors with mismatch repair-deficient (dMMR) or microsatellite-instability-high (MSI-H). In this subset of patients it is found a large number of activated CD8-positive cytotoxic T cells and upregulated checkpoints (e.g., CTLA 4, PD-1, and/or PD-L1) (13, 14).

Besides dMMR/MSI-H patients, other subjects who respond to PD-1/PD-L1 inhibitors have tumors with an even higher TMB known as hypermutated tumors, e.g., tumors harboring polymerase proofreading domain mutations (POLE), which are thought to also be susceptible to immunotherapy (15, 16).

dMMR CRCs are found in 15–20% of stage II and III CRCs and are associated with a better prognosis than proficient mismatch repair (pMMR) tumors. In the metastatic setting, dMMR CRCs represent only around 5% and are associated with a worse prognosis (17).

Researches showed that most CRC patients are not responsive to immunotherapy. Several factors may be involved in this lack of sensitivity. Lack of T-cell infiltration, type 1 T-helper cell activity and low immune cytotoxicity in tumor microenvironment are thought to be involved (18, 19). Many attempts have been conducted to characterize CRC and detect subgroups susceptible for individual treatments [e.g., the four consensus molecular subtypes (CMS 1-4)]. CMS 1 (immune) subtype already specifies high TMB subgroup, which is responsive to immunotherapy (20). Although the CMS classification may help to guide clinicians and researchers in management and treatment of CRC, there is no clinical relevance yet (21).

## Evidence Acquisition

**Figure 2:**
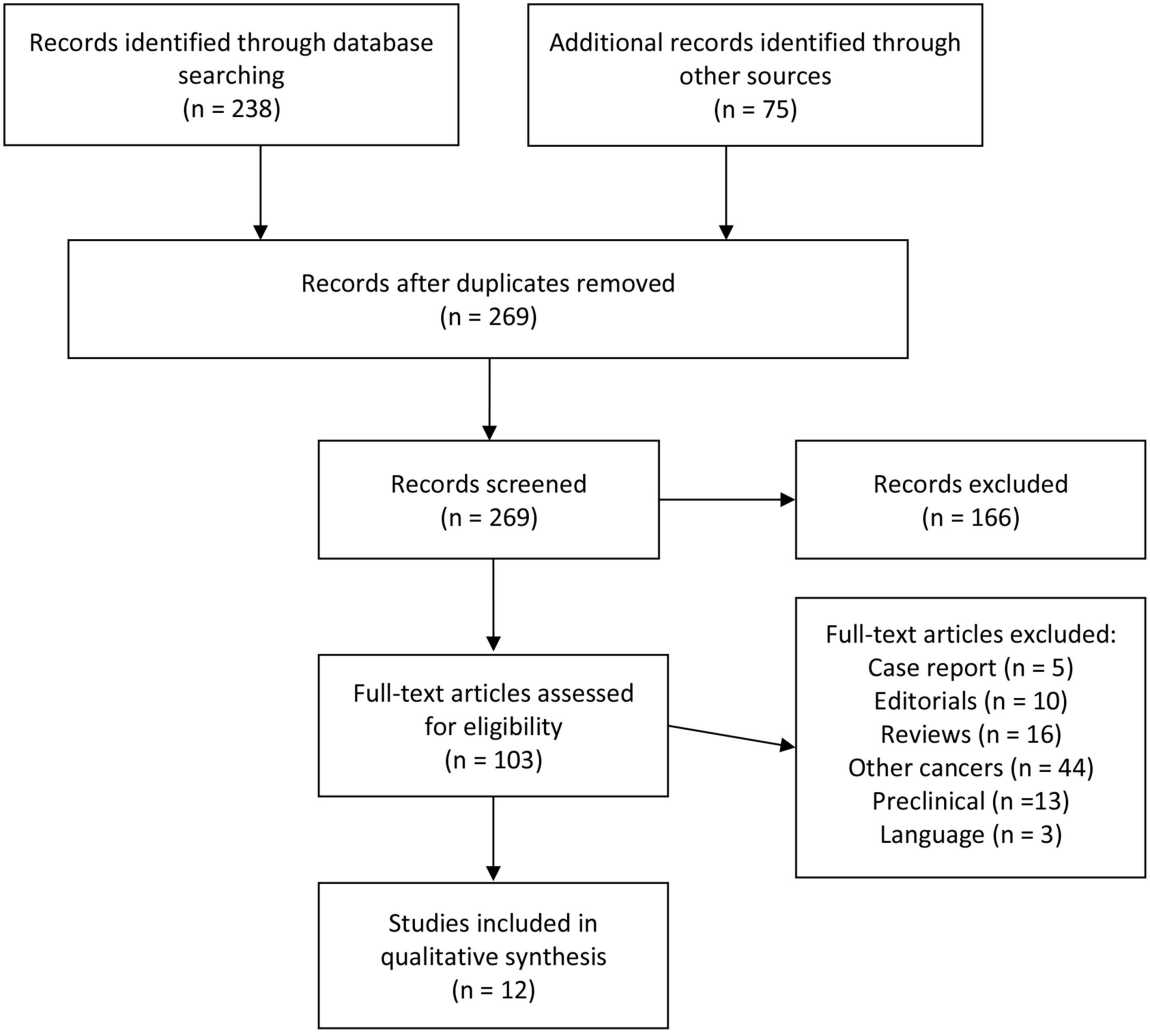
PRISMA flow chart of study selection.

An article search was performed in PubMed and Cochrane databases to identify clinical and randomized controlled trials published until August 2018. Multiple algorithms that included the following terms were used: colorectal cancer, colon cancer, immunotherapy, PD-1, PD-L1, MSI-H, dMMR, and microsatellite instability. Inclusion criteria used were published full articles, clinical trials, retrospective series, meta-analyses, and English language. Since immunotherapy is a very active research field, we also explored abstracts from known oncology conferences during 2014–2018. Our systematic review focused on published clinical trials according to Preferred Reporting Items for Systematic Reviews and Meta-analyses (PRISMA) guidelines (22). The flow diagram for article selection is shown in Figure 2. Each identified article was analyzed and classified. Primary outcomes comprised oncologic results with progression free and/or overall survival and tumor response rates. We also recorded and reviewed ongoing clinical trials that are in progress within the subject of the review. The search was done mainly on clinicaltrials.gov with the same algorithms described above.

## Results

### Single-Agent PD-1 Inhibitor

#### Pembrolizumab

The first study to show the clinical efficacy of PD-1 blockade in the microsatellite instability subset of colorectal cancer patients was with Pembrolizumab, an Anti-PD1 inhibitor. The trial examined the treatment of tumors with MMR deficiency following a recurrence of disease after standard treatments in a wide range of malignancies. Clinical activity of pembrolizumab 10 mg/kg was evaluated in 3 patient groups: those with dMMR mCRC, pMMR and a third group that included patients with dMMR in other malignancies (non-CRC) (13). Overall response rates were 40% (4 of 10 patients) with a 78% progression-free survival rate at 20 weeks (7 of 9 patients) in the cohort of dMMR mCRC. In the second group of those with pMMR, the objective response rate was 0% (0 of 18 patients), with only 11% exhibiting Progression-Free Survival (PFS) at 20 weeks. These results highlighted the activity of Pembrolizumab in this subgroup of patients with dMMR mCRC, leading to the approval of the first-ever agnostic (i.e., histology and tumor-site independent) cancer drug in May 2017 in the US.

A second phase Ib trial, the KEYNOTE-028, evaluated Pembrolizumab in PD-L1–positive advanced solid tumors. The results for the CRC cohort were published (23). Patients with advanced and treatment-resistant PD-L1–positive were enrolled, disregarding microsatellite status. Pembrolizumab in a dose of 10 mg/kg was administered every 2 weeks for up to 2 years or until disease progression/unacceptable toxicity. Primary endpoints were safety and overall response rate. 23 patients were enrolled with a median follow-up of 5.3 months. Most patients (n = 15, 65%) experienced progressive disease. One partial response occurred in a patient (4%) with MSI-H CRC. Pembrolizumab demonstrated a favorable safety profile and its antitumor activity was only observed this single patient with MSI-H. This justified an evaluation for its use in this patient population.

KEYNOTE-164 was another study with Pembrolizumab. The cohort included patients previously treated with more than one line of therapy with mCRC MSI-H (24). Patients received pembrolizumab 200 mg every 3 weeks. Endpoints included objective response rate (ORR) (primary) and duration of response (DOR), PFS, overall survival (OS). Sixty-three patients were enrolled. The most recent results published an ORR of 32%, two complete responses and 18 partial responses. Median PFS was 4.1 months and median OS was not reached. The 12-month PFS rate was 41%, and the 12-month OS rate was 76%. This study revealed a durable antitumor in patients with MSI-H CRC treated with Pembrolizumab who progressed after a first-line of therapy. These findings are currently being evaluated in a larger phase III trial, the KEYNOTE-177, in patients with dMMR mCRC (25).

#### Nivolumab

Nivolumab was initially studied in phase I trial on various solid tumors. Only 14 patients enrolled had mCRC. A durable complete response was reported on a single patient. Nivolumab was further evaluated in a phase II trial on 296 patients but no objective responses were achieved among those with mCRC. As the only responder was found to have a deficient MMR (dMMR) tumor, this status was presumed to be a predictor of efficacy (10).

A phase 2 trial, CheckMate 142, provided further evidence for the use of Nivolumab in dMMR/MSI-H mCRC (26). Seventy four patients recruited were assessed locally with dMMR/MSI-H CRC. Seventy two percent were confirmed after central molecular testing to have MSI-H tumors. Primary endpoint was an investigator-assessed overall response (iORR), achieved in 23 patients (31,1%). Durable responses (≥12 months) were reported in eight (35%) of 23 responders. PFS and OS at 1 year were 50 and 73%, respectively. An improvement in some parameters quality of life was also documented.

### Combinations With PD-1/PD-L1 Inhibitors

The large population of mCRC patients are not dMMR/MSI-H which make up to 95% of patients. Based on previously reported low efficacy of immunotherapy in unselected patients, several combination regimens with local ablation, chemotherapy or molecularly targeted drugs have been already evaluated (Table 1) and a large number of trials in this setting are still ongoing (Tables 2, 3) either in selected or unselected populations.

**Table 1:** Results from clinical trials with PD-1/PD-L1 inhibitors.

**Study/ClinicalTrials.gov Identifier**	**Drug(s)**	**N**	**Patient population**	**(iO)RR**	**Phase**	**Primary endpoint**	**12m OS**
Le et al. (13), NEJM 2015 NCT01876511	Pembrolizumab	41 (32 CRC)	dMMR:11 pMMR 21	dMMR 40% pMMR 0%	II	irPFS	–
Lee et al. (27), JCO 2017 NCT02260440	Pembrolizumab + azacitidine	31	30 pts with MSS mCRC	3%	II	ORR	–
Shahda et al. (28), JCO 2017 NCT02375672	Pembrolizumab + mFOLFOX6	30 (3 MSI-H)	1st line mCRC	53%	II	mPFS	–
O'Neil et al. (23), BH 2017 NCT02054806	Pembrolizumab	137 (23 enrolled)	PD-L1 positive refractory mCRC	4%	Ib	ORR	29,8%
Le Dung et al. (24), KEYNOTE-164 NCT02460198	Pembrolizumab	63	MSI-H mCRC treated with ≥1 prior line	32%	II	ORR	76%
NCT02788279	Atezolizumab +- Cobimetinib	363 (1.7% MSI-H)	MSS/MSI-L mCRC	2,7%	III	OS	–
NCT01633970	Atezolizumab + FOLFOX + Bevacizumab	23	Refractory mCRC	52%	Ib	Safety	–
Brahmer et al. (10), NEJM 2012 NCT00729664	Nivolumab	19	mCRC MSI unknown	0%	I (multi tumors)	Safety	–
CheckMate142 NCT02060188	Nivolumab	74	dMMR/MSI-H mCRC	31,1%	II	ORR	85%
CheckMate142 NCT02060188	Nivolumab + Ipilimumab (4 doses)	119	dMMR/MSI-H refractory mCRC	55%	II	ORR	85%
CheckMate142 NCT02060188	Nivolumab + Ipilimumab (1mg/kg) Q6W	45	dMMR/MSI-H First-line mCRC	60%	II	ORR	83%
NCT02298946	CTX + AMP-224 + SBRT	17	mCRC	0%	I	Safety	–

*CTX, cyclophosphamide; SBRT, stereotactic body radiation therapy; mCRC, metastatic colorectal cancer; MSI, microsatellite instability; H, high; MSS, microsatellite stability; pMMR, mismatch repair proficient; ORR, objective response rate; irORR, immune-related ORR; PFS, progression-Free Survival; OS, overall survival; RR, response rate; BRR, best RR. Details available at: www.clinicaltrials.gov*.

**Table 2:** Ongoing Phase II and III trials with PD-1/PD-L1 inhibitors.

**ClinicalTrials.gov identifier**	**Drug(s)**	**Phase**	**Patient Population**	**Primary Endpoint**	**Completion Date**
NCT03396926	Pembrolizumab + bevacizumab + capecitabine	II	pMMR mCRC	ORR	April 2021
NCT03259867	TATE treatment + Pembrolizumab	IIA	Liver metastasis from CRC	RR	October 2021
NCT03519412	Induction (pMMR): Temozolomide Treatment: Pembrolizumab	II	dMMR or pMMR mCRC	ORR	July 2019
NCT03631407	Vicriviroc + Pembrolizumab	II	MSS mCRC	ORR	March 2025
NCT02981524	CY/GVAX with Pembrolizumab	II	MMR-p mCRC	ORR	November 2017
NCT02563002	Pembrolizumab	III	MSI-H/dMMR mCRC	PFS, OS	March 2025
NCT02437071	Pembrolizumab + RT	II	pMMR mCRC	ORR	September 2019
NCT02227667	Durvalumab	II	mCRC MSI-H	BRR	August 2021
NCT02870920	Durvalumab + Tremelimumab	II	Refractory mCRC	OS	February 2019
NCT02997228	Atezolizumab +- (Bevacizumab + mFOLFOX6)	III	dMMR mCRC	PFS	March 2022
NCT02873195	Atezolizumab + Capecitabine + Bevacizumab	II	Refractory mCRC	PFS	November 2022
NCT02291289	Atezolizumab	II	mCRC	PFS	April 2019
NCT02992912	Atezolizumab + SABR	II	Metastatic multi tumors	PFS	December 2021
NCT03050814	Avelumab + vaccine Ad-CEA	II	mCRC	PFS	November 2020
NCT03186326	Avelumab	II	Second line MSI-H mCRC	PFS	December 2018
NCT03642067	Nivolumab + Relatlimab	II	MSS mCRC	ORR	November 2021
NCT02860546	Nivolumab + TAS 102	II	mCRC MSS	irORR	November 2017
NCT03638297	BAT1306 + Cox inhibitor	II	MSI-H/dMMR or High TMB	RR	January 2023

*mCRC, metastatic colorectal cancer; MSI, microsatellite instability; MSS, microsatellite stability; pMMR, mismatch repair proficient; ORR, objective response rate; irORR, immune-related ORR; PFS, progression free survival; OS, overall survival; RR, response rate; BRR, best RR. Details available at: www.clinicaltrials.gov*.

**Table 3:** Ongoing Phase I and II trials with PD-1/PD-L1 inhibitors.

**ClinicalTrials.gov identifier**	**Drug(s)**	**Phase**	**Patient Population**	**Primary Endpoint**	**Completion Date**
NCT02851004	BBI608 (Napabucasin) + Pembrolizumab	Ib/II	mCRC	irORR	June 2022
NCT03531632	MGD007 + MGA012	I/II	mCRC	Safety	December 2019
NCT03274804	Maraviroc + Pembrolizumab	I	MSS mCRC	Safety	April 2022
NCT03374254	Pembrolizumab + Binimetinib (+-CT)	I	mCRC	Safety	November 2019
NCT03202758	Durvalumab + Tremelimumab + FOLFOX	Ib/II	Refractory mCRC	–	October 2022
NCT02437136	Entinostat + Pembrolizumab	Ib/II	pMMR mCRC	–	August 2020
NCT02636036	Enadenotucirev + Nivolumab	I	Metastatic or advanced epithelial tumors	Safety	August 2019
NCT02777710	Pexidartinib + Durvalumab	I	Metastatic/advanced pancreatic or colorectal cancers	Safety	March 2020
NCT03206073	Durvalumab + Pexa-Vec +- Tremelimumab	I/II	Refractory mCRC	PFS	June 2019
NCT03332498	Ibrutinib + Pembrolizumab	I/II	Refractory mCRC	Safety	December 2021
NCT02886897	D-CIK and anti-PD-1 antibody	I/II	Multi tumors	PFS	February 2022
NCT02335918	Varlilumab + Nivolumab	I/II	Multi tumors	ORR	October 2019
NCT03058289	INT230-6 + Pembrolizumab	I/II	Multi tumors	Safety	May 2020
NCT02834052	Pembrolizumab + Poly-ICLC	I/II	pMMR CRC	RR	August 2020
NCT02959437	Pembrolizumab + Epacadostat + (Azacitidine or INCB057643)	I/II	MSS mCRC	ORR	January 2021
NCT03085914	Epacadostat + Pembrolizumab + mFOLFOX6	I/II	Advanced or metastatic solid tumors	ORR	October 2020
NCT02903914	INCB001158 + Pembrolizumab	I/II	Multi tumors	Safety	October 2022
NCT03168139	Olaptesed pegol + Pembrolizumab	I/II	Refractory mCRC	Safety	May 2019
NCT02650713	RO6958688 + Atezolizumab	Ia/Ib	Refractory mCRC	Safety	July 2019

*mCRC, metastatic colorectal cancer; MSS, microsatellite stability; pMMR, mismatch repair proficient; ORR, objective response rate; irORR, immune-related ORR; PFS, progression-free survival. Details available at: www.clinicaltrials.gov*.

The combination of immune checkpoint inhibitors with Nivolumab and Ipilimumab (anti-CTLA4) in dMMR/MSI-H mCRC patients were studied in a cohort with 119 patients of the CheckMate 142. Published outcomes demonstrated a consistent clinical effect with an ORR of 55% and a 12-weeks disease control rate-rate 80% (29). Responses were durable with a PFS rate of 71% and OS of 85% after 1 year. Responses were independent RAS/BRAF mutation status, PD-L1 expression or Lynch syndrome history. Patients recruited were heavily pre-treated with majority having at least two prior lines of therapy for metastatic disease. Recently published, is another cohort of the same study but in first-line chemorefractory mCRC with nivolumab plus low dose ipilimumab. It resulted in lower toxicity and with a median of 2.6 months for patients to respond to treatment. The ORR was 60%, the disease control rate was 84%, and 7% of patients had a complete response (30).

Other studies combining Pembrolizumab with chemotherapy were published. Pembrolizumab plus Azacytidine was evaluated in a phase 2 trial to assess anti-tumor activity and safety in patients with previously treated mCRC without standard treatment options. The combination showed a tolerable safety profile but had a little anti-tumor effect for MSS mCRC (27). Pembrolizumab was also tested with mFOLFOX6 in a phase 2 trial of untreated or unresectable CRC. 30 patients were assigned to a single arm study with 3 of them being MSI-H. Treatment resulted in 15 patients achieving partial responses (CR+PR = 53%) and 14 stable diseases. Of note, is a single patient with dMMR that had resection that showed complete pathologic response after 2 months of therapy. Increased neutropenia was the main toxicity found. Clinical activity was identified, including those with pMMR (28).

The combination of Atezolizumab an anti–PD-L1 monoclonal antibody with FOLFOX and Bevacizumab was studied in a multi tumor phase I trial. Preliminary data suggests that the combination can promote immune-related activity resulting in enhanced efficacy. However, more robust data is need (31).

Atezolizumab in association with Cobimetinib a MEK1/MEK2 inhibitor in the MAPK pathway was also studied in patients with MSS/MSI-L mCRC in the IMblaze370 trial. The results of primary analysis were presented recently. The study did not however meet its primary endpoint which was OS with a median of 8.9 month with combination vs. 8.5 month with regorafenib. In this study, almost all patients included (91.7%) had tumors with MSS/MSI-L status (32).

Lastly, PD-L2 Fc fusion protein that binds to PD-1, known as AMP-224, was tested in combination with stereotactic body radiation therapy (SBRT) plus cyclophosphamide in mCRC with hepatic metastasis. This combination appeared safe and feasible, but preliminarily data showed no objective responses. Expected in a near future are clinical and correlative data including post-therapeutic radiated and non-radiated tumor biopsies (33).

## Discussion and Challenges

Many of the published studies are early phase clinical trials, with limited number of mCRC patients recruited. At least six trials with favorable objective response and improved progression-free survival in patients with MSI-H CRCs were observed. These results are encouraging. However, the population of dMMR/MSI-H is very low, representing only 5% of patients in the metastatic setting. ORR in CheckMate142 and KEYNOTE-164 was around 30% with a substantially higher rate 52% reported in KEYNOTE-016; 1-year overall survival rates were very high which may indicate durable responses. (26).

The clinical trials with PD-1/PD-L1 inhibitors also revealed different results according to the patient population studied. Outcomes in MSS/pMMR population including a phase III study with Atezolizumab plus Cobimetinib were negative, not providing any evidence of its use in this context.

In non-metastatic CRC, dMMR accounts for approximately 15–20% which can be found more frequently. An adjuvant trial with an anti-PD-L1 is ongoing, but it remains unknown its effectiveness in this situation. Adjuvant ipilimumab or nivolumab have shown to extend survival in stage III or IV (resected) but mechanisms of resistance in CRCs in this setting are not necessarily the overexpression of immune checkpoints (34).

Particular patients may present primary or secondary resistance to immune checkpoint inhibitors. It will be important in the future to study mechanisms of primary and acquired resistance to PD-1 blockade. Furthermore, there are many rationales for the study of combination therapies with PD-1 inhibitors and compounds targeting other immune regulators (e.g., CTLA-4, LAG-3, OX-40, TIM-3, KIR, VISTA, GITR, IDO-1,2, and others). They might prove together more effective or have other properties after certain resistances. The highlighted any many other strategies involving targeted therapy, chemotherapy and radiotherapy are ongoing with the aim to enhance response to immunotherapy (Tables 2, 3).

Current predictive biomarkers for the efficacy of anti PD1 are dMMR status and POLE mutations. POLE mutations are associated with an ultramutated phenotype and are reported in 1–2% of colorectal cancers (35). Still a subset of patients with immune-sensitive tumors, can be probably identified with molecular subtyping, immune-inflammation gene expression signature or immunoscoring (20, 35). Lastly, gut microbiome modulation and other therapy combinations might prove to beneficial and enhance the effects of immunotherapy in under other conditions resistant tumors (18, 36).

The challenges pointed out, and many others are ahead and will include other clinical and feasibility issues. Questions like how to increase or reinforce the efficacy of immunotherapy, the optimal duration and combinations of treatments (37) and indications of surgical interventions after immunotherapy (38) will be slowly unraveled. Also, feasibility will undoubtedly be discussed since the higher costs of this drugs can add a substantial burden to healthcare systems, as the example of Canada (39).

## Conclusions

The outcome of CRC patients has improved considerably over the past two decades. The efficacy of systemic therapies and biomarker-based treatments has been predominant in this positive change. A better understanding of the interaction between a tumor and the immune system in the last decade led to the development of new agents, in particular, the PD-1/PD-L1 inhibitors.

Even though PD-1 inhibitors have shown efficacy in dMMR CRCs, there are still many questions which need to be answered, e.g. how could we conceive a response on pMMR CRCs and how to find predictive biomarkers of efficacy. dMMR/MSI-H tumors only account for a small 5% of mCRC. There is no doubt that extending the benefit of immunotherapy to a broader, microsatellite stable population would be the next but nevertheless difficult step.

Many trials are already in progress exploring combinations strategies. Other important topics that in the future will be important to address are the role of immunotherapy and anti-PD1 therapy in the prevention of CRC, conversion therapy of potentially resectable liver metastases, and adjuvant or neoadjuvant treatment. Trials already published have been received by gastrointestinal oncology community with great enthusiasm, and future studies with PD-1 inhibitors mCRC will further help us to decipher the many pieces of a big puzzle with immunotherapy.

## Author Contributions

All authors listed have made a substantial, direct and intellectual contribution to the work, and approved it for publication.

### Conflict of Interest Statement

The authors declare that the research was conducted in the absence of any commercial or financial relationships that could be construed as a potential conflict of interest.
